# Long Noncoding RNA HCG11 Acts as a Tumor Suppressor in Gastric Cancer by Regulating miR-942-5p/BRMS1 Axis

**DOI:** 10.1155/2021/9961189

**Published:** 2021-05-11

**Authors:** Qingmei Zhang, Keli Yang, Jie Li, Fang Chen, Yan Li, Qiuju Lin

**Affiliations:** ^1^Emergency Ward, Qingdao Hospital of Traditional Chinese Medicine, Qingdao Hiser Hospital, Qingdao 266033, China; ^2^Gastroenterology Department, People's Hospital of Chiping, Chiping 252100, China; ^3^Department of Oncology, People's Hospital of Rizhao, Rizhao 276800, China; ^4^ICU, the People's Hospital of Zhangqiu Area, Jinan 250200, China; ^5^Interventional Vascular Department, the People's Hospital of Zhangqiu Area, Jinan 250200, China; ^6^Oncology Department (II), Qingdao Central Hospital, Qingdao University, Qingdao 266042, China

## Abstract

The functions of long noncoding RNAs (lncRNAs) have been widely investigated in human cancers, including gastric cancer (GC). The purpose of this study was to elucidate the role of lncRNA HCG11 in GC. In this study, mRNA and protein expressions were detected by quantitative real-time polymerase chain reaction assays (RT-qPCR) and Western blot analysis. The proliferation ability of GC cells was examined by (3-(4,5-Dimethylthiazol-2-yl)-2,5-Diphenyl Tetrazolium Bromide) MTT assays. The invasion and migration abilities of GC cells were evaluated by Transwell assays. The binding sites between miR-942-5p and HCG11/BRMS1 were confirmed by dual-luciferase reporter assays. Results showed that LncRNA HCG11 was downregulated in GC cells. Functionally, overexpression of HCG11 inhibited GC cell proliferation, migration, and invasion. In addition, lncRNA HCG11 was found to act as a molecular sponge of miR-942-5p. Furthermore, miR-942-5p promoted GC progression by suppressing lncRNA HCG11 expression. Besides that, BRMS1 was confirmed as a direct target of miR-942-5p. More importantly, breast cancer metastasis suppressor 1 (BRMS1) inhibited GC progression by upregulating lncRNA HCG11 and downregulating miR-942-5p. In conclusion, LncRNA HCG11 inhibited cell proliferation, migration, and invasion in GC by sponging miR-942-5p and upregulating BRMS1.

## 1. Introduction

Gastric cancer (GC) is a malignant tumor originating from the gastric mucosal epithelium, and its incidence has been increasing in recent years. Moreover, GC not only causes damage to the digestive system but also may cause metastasis, affecting liver, kidney, and respiratory function [1]. Previous studies have shown that the tumorigenesis of GC may be related to smoking, high salt intake, viral infection, and heredity [2]. Although the current medical technology has made significant progress, the five-year overall survival rate of GC patients in China is still not optimistic, maintaining around 30% [3]. Recently, targeted therapy has become a hot spot for the treatment of cancer, including GC. Therefore, finding effective therapeutic targets is of great significance for improving the survival rate of GC patients.

As widely recognized targets, long noncoding RNAs (lncRNAs) have been reported to act as tumor suppressors and oncogenes in human cancers [4]. In addition, the functions of lncRNAs have been identified in GC [5]. For example, upregulation of lncRNA AWPPH inhibited proliferation and invasion of GC cells via miR-203a/DKK2 axis [6]. On the contrary, lncRNA SNHG8 promoted proliferation and invasion of GC cells by targeting the miR-491/PDGFRA axis [7]. Recently, the specific roles of lncRNA HCG11 in other cancers caught our attention. Low expression of lncRNA HCG11 has been found in glioma and prostate cancer. Furthermore, lncRNA HCG11 inhibited the progression of glioma and prostate cancer [8, 9]. However, the dysregulation of HCG11 in GC remains unrevealed and needs to be illuminated. To further explore the regulatory mechanism of HCG11 in GC, miR-942-5p was predicted to be a downstream regulator of HCG11.

Previous studies have reported that miR-942-5p can be involved in the pathogenesis of human cancers. Upregulation of miR-942-5p has been detected in Huntington's disease and Kaposi's sarcoma and metastatic renal cell carcinoma [10, 11]. Functionally, miR-942-5p has been reported to promote proliferation and metastasis of hepatocellular carcinoma cells by inhibiting RRM2B [12]. More importantly, lncRNA Linc00675 suppressed cell proliferation and metastasis in colorectal cancer via targeting miR-942 [13]. These findings suggest that miR-942-5p acts as a tumor promoter in cancers. However, little is known about the regulatory mechanism of lncRNA HCG11/miR-942-5p in GC. Besides that, it is well known that miRNAs regulate human cancers by interacting with target genes. In this study, breast cancer metastasis suppressor 1 (BRMS1) was found to have a binding site with miR-942-5p.

BRMS1 was initially identified in breast carcinoma and suppressed breast cancer metastasis [14]. Recently, the function of BRMS1 has been identified in other cancers. For example, BRMS1 can suppress metastasis and correlate with improved patient survival in non-small cell lung cancer [15]. Besides, BRMS1 can coordinately regulate miRNA expression to participate in tumorigenesis [16]. For example, Guo *et al.* reported that miR-346 promoted hepatocellular carcinoma progression by suppressing BRMS1 expression [17]. In particular, downregulation of BRMS1 has been found in GC tissues [18]. Nevertheless, the function of BRMS1 in GC remains largely unknown.

Therefore, we explored the role of BRMS1 as well as its interaction with lncRNA HCG11/miR-942-5p in GC. Simultaneously, the regulatory mechanism of lncRNA HCG11/miR-942-5p was also investigated in GC. This study will provide a novel regulatory network in GC.

## 2. Materials and Methods

### 2.1. Cell Culture

Normal human gastric epithelium cell line GES-1 and GC cell line AGS and HGC-27 were purchased from BeNa Culture Collection (BNCC, Beijing, China). These cells were seeded in RPMI-1640 medium with 10% FBS and incubated in a humid atmosphere with 5% CO_2_ at 37°C.

### 2.2. Cell Transfection

The pcDNA3.1 vector containing HCG11 complementary DNA, HCG11 siRNA, BRMS1 siRNA, miR-942-5p mimic, and miR-942-5p inhibitor were purchased from GenePharma (Shanghai, China). Next, they were transfected into AGS cells using Lipofectamine 2000 (Invitrogen/Thermo Fisher Scientific).

### 2.3. RT-qPCR

Total RNA extraction was performed using TRIzol reagent (Invitrogen, USA). The cDNA solution was synthesized using a PrimeScript RT reagent kit (Takara, Dalian, China). RT-qPCR assay was performed on ABI 7300 real-time PCR system (Applied Biosystems, Waltham, MA) using SYBR Green Master Mix II (Takara). HCG11 and miR-942-5p expression were normalized to U6, while BRMS1 was normalized to GAPDH. Their expressions were quantified with the 2^−△△cq^ method. The primers used were as follows: HCG11 forward 5′-AGG AGT GGT TGC ATT TGG GA-3′′; HCG11 reverse 5′′-CCC ACC ACG CAG TGA ATA GT-3′; miR-942-5p forward: 5′-GCC AGA TCT TGA TTG ACT TAC AGC CCA GTT-3′ and reverse, 5′-GCC GAA TTC CAC CTG TCT TTA TTC CAC CC-3′; U6-forward: 5′-GCT TCG GCA GCA CAT ATA CTA AAA T-3′ and reverse, 5′-CGC TTC ACG AAT TTG CGT GTC AT-3′; BRMS1 forward: 5′-CAG CCT CCA AGC AAA GAC AC-3′ and reverse, 5′-GCG GCG TCG CTC ATA GTC-3′; GAPDH forward: 5′-ACA ACT TTG GTA TCG TGG AAG G-3′, and reverse, 5′-GCC ATC ACG CCA CAG TTT C-3′.

### 2.4. MTT Assay

Transfected AGS cells (3 × 10^3^ cells/well) were incubated in RPMI-1640 medium containing 10% FBS for 24, 48, 72, or 96 h. Then, AGS cells were incubated with 20 *μ*l of MTT for 4 h. Next, 150 *μ*l of dimethyl sulfoxide was added to the medium. After 10 minutes, we evaluated the cell viability using a spectrophotometer (Olympus Corp., Tokyo, Japan) to determine the optical density at 490 nm.

### 2.5. Transwell Assay

Cell invasion was detected in the upper chamber with Matrigel. Cell migration experiment was performed without Matrigel. Next, AGS cells (3 × 10^3^ cells/well) were added to Transwell upper chamber. RPMI-1640 medium with 10% FBS was added to the lower chamber. After 24 h, the moving cells were fixed and stained for 30 mins. Observation and photographing were performed by a light microscope.

### 2.6. Western Blot Analysis

The protein was lysed using RIPA lysis buffer (Beyotime, Shanghai, China). Next, the protein was electrophoresed by 10% SDS-PAGE. Protein samples were blocked with 5% nonfat milk and transferred into PVDF membranes. Protein samples were then incubated with E-cadherin, N-cadherin, Bcl-2, Bax, and GAPDH primary antibodies (Abcam, Shanghai, China) overnight at 4°C. Secondary antibodies were added to incubate the protein for 1 h. Finally, the protein was examined using an ECL kit (Beyotime).

### 2.7. Dual-Luciferase Reporter Assay

The 3′-UTR of wild-type and mutant HCG11 (wt-HCG11 and mut-HCG11) or BRMS1 (wt-BRMS1 and mut-BRMS1) was inserted into pmiR-GLO vector (Promega Beijing Biotech Co., Beijing, China). Next, the above reporter plasmids or miR-942-5p mimics were transfected into AGS cells. After 48 h, luciferase activities were determined by a dual-luciferase reporter assay system (Promega, USA).

### 2.8. Statistical Analysis

Data are shown as mean ± SD and analyzed using Student's *t*-test or one-way ANOVA in SPSS 19.0 or Graphpad Prism 6. *P* < 0.05 was defined as a statistical difference.

## 3. Results

### 3.1. LncRNA HCG11 Inhibits Cell Proliferation, Migration, and Invasion in GC

**Figure 1:**
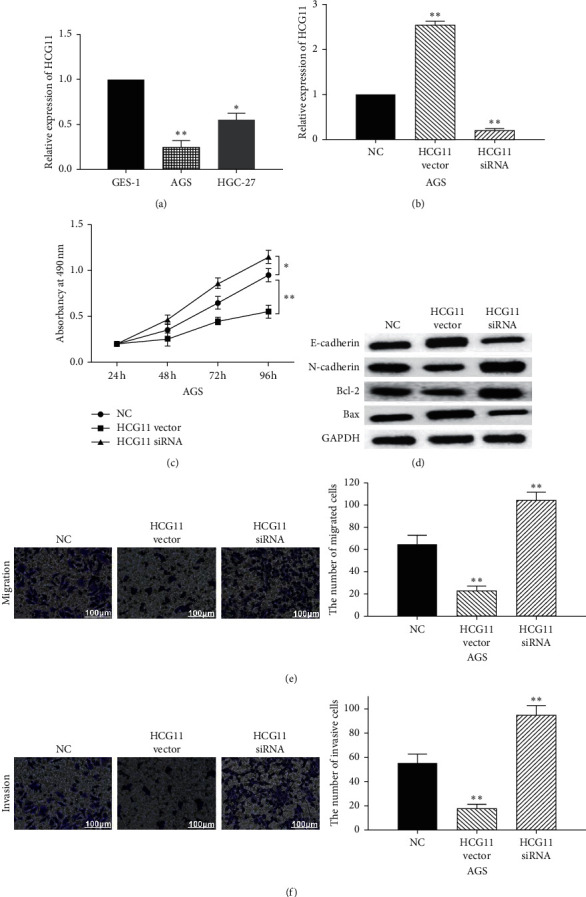
LncRNA HCG11 inhibits cell proliferation, migration, and invasion in GC. (a) The expression of HCG11 was detected in GES-1, AGS, and HGC-27 cells. (b) LncRNA HCG11 expression in AGS cells with its vector or siRNA. (c) Cell proliferation in AGS cells with HCG11 vector or siRNA. (d) The protein expressions of E-cadherin, N-cadherin, Bax, and Bcl-2 in AGS cells with HCG11 vector or siRNA. (e, f) Cell migration and invasion in AGS cells with HCG11 vector or siRNA. ^*∗*^*P* < 0.05,  ^*∗∗*^*P* < 0.01.

First, the expression of HCG11 was detected in GES-1, AGS, and HGC-27 cells. RT-qPCR showed that HCG11 was downregulated in AGS and HGC-27 GC cells compared to GES-1 cells (Figure 1(a)). Furthermore, the expression of HCG11 is significantly decreased in AGS cells compared to HGC-27 cells. Thus, AGS cells were used to explore the role of HCG11 in GC. Next, HCG11 vector or siRNA was transfected into AGS cells, respectively. We found that HCG11 expression was inhibited by HCG11 siRNA and promoted by HCG11 vector (Figure 1(b)). MTT assay indicated that overexpression of HCG11 inhibited cell proliferation, while knockdown of HCG11 promoted cell proliferation in AGS cells (Figure 1(c)). Additionally, HCG11 vector promoted E-cadherin and Bax expression and inhibited N-cadherin and Bcl-2 expressions, while HCG11 siRNA showed an opposite effect on these genes in AGS cells (Figure 1(d)). Next, the Transwell assay showed that cell migration and invasion were restrained by HCG11 overexpression and promoted by HCG11 siRNA (Figures 1(e) and 1(f)). These results imply that lncRNA HCG11 inhibits cell proliferation, migration, and invasion in GC.

### 3.2. LncRNA HCG11 Acts as a Molecular Sponge of miR-942-5p

**Figure 2:**
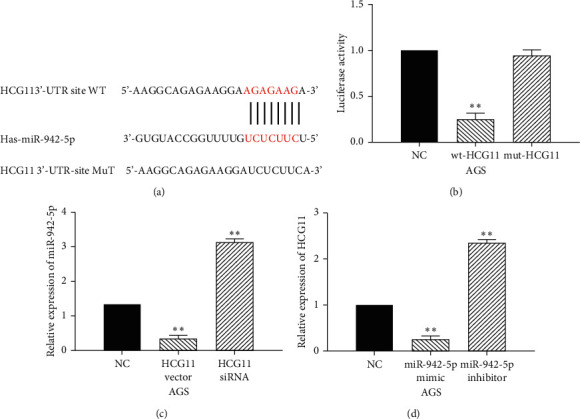
LncRNA HCG11 acts as a molecular sponge of miR-942-5p. (a) The binding sites of HCG11 with miR-942-5p. (b) Luciferase reporter assay. (c) MiR-942-5p expression regulated by HCG11 siRNA and vector in AGS cells. (d) HCG11 expression in AGS cells containing miR-942-5p mimics or inhibitor. ^*∗∗*^*P* < 0.01.

Next, starBase version 2.0 (http://starbase.sysu.edu.cn/) predicts that lncRNA HCG11 has a binding site with miR-942-5p (Figure 2(a)). Besides, the dual-luciferase reporter suggested that miR-942-5p mimics reduced the luciferase activity of wt-HCG11 but had little effect on mut-HCG11 luciferase activity in AGS cells (Figure 2(b)). Next, miR-942-5p expression was examined in AGS cells with HCG11 siRNA and vector. We found that HCG11 overexpression decreased miR-942-5p expression, while HCG11 downregulation promoted miR-942-5p expression in AGS cells (Figure 2(c)). Meanwhile, HCG11 expression was detected in AGS cells with miR-942-5p mimics or inhibitors. HCG11 expression was found to be reduced by miR-942-5p mimics and enhanced by a miR-942-5p inhibitor (Figure 2(d)). Based on these results, lncRNA HCG11 was considered to act as a molecular sponge of miR-942-5p.

### 3.3. MiR-942-5p Is Involved in GC Progression by Mediating lncRNA HCG11

**Figure 3:**
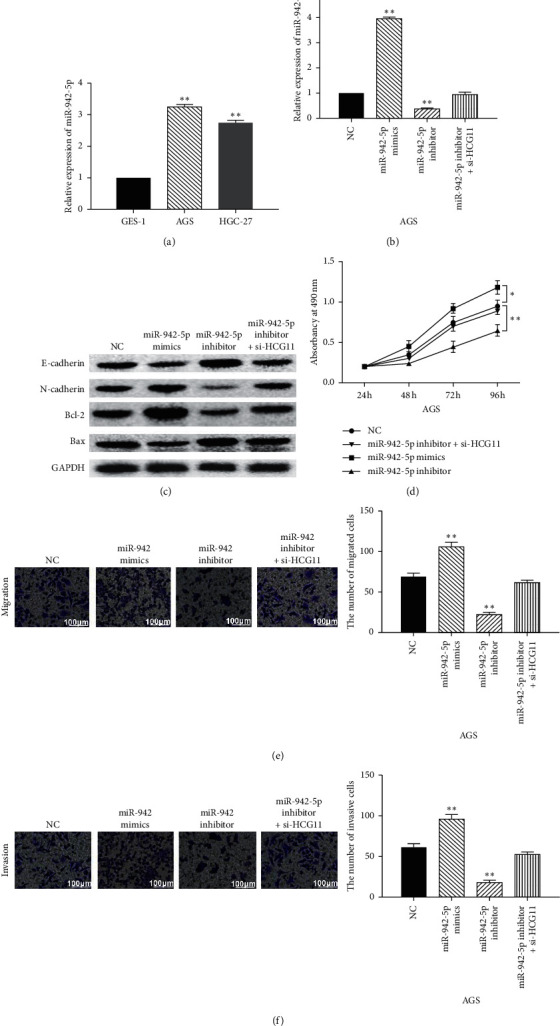
MiR-942-5p is involved in GC progression by mediating lncRNA HCG11. (a) The expression of miR-942-5p was detected in GES-1, AGS, and HGC-27 cells. (b) MiR-942-5p expression in AGS cells with miR-942-5p mimics, miR-942-5p inhibitor, or HCG11 siRNA. (c) The protein expressions of E-cadherin, N-cadherin, Bax, and Bcl-2 in AGS cells with miR-942-5p mimics, miR-942-5p inhibitor, or HCG11 siRNA. (d, e, f) Cell proliferation, migration, and invasion in AGS cells with miR-942-5p mimics, miR-942-5p inhibitor, or HCG11 siRNA. ^*∗*^*P* < 0.05,  ^*∗∗*^*P* < 0.01.

The expression of miR-942-5p was detected in GC cells. We found that miR-942-5p was upregulated in AGS and HGC-27 GC cells compared to GES-1cells (Figure 3(a)). To explore the interaction between miR-942-5p and lncRNA HCG11, miR-942-5p mimics, miR-942-5p inhibitor, or HCG11 siRNA was transfected into AGS cells. RT-qPCR showed that miR-942-5p mimics enhanced its expression, whereas miR-942-5p inhibitor decreased its expression in AGS cells. However, HCG11 siRNA recovered the decreased expression of miR-942-5p induced by its inhibitor (Figure 3(b)). Additionally, miR-942-5p mimics were found to promote N-cadherin and Bcl-2 expressions and suppress E-cadherin and Bax expression, while miR-942-5p inhibitor exerted the opposite effect on these genes. HCG11 siRNA exerted a reverse effect on the expression of these genes regulated by miR-942-5p inhibitor in AGS cells (Figure 3(c)). Functionally, cell proliferation, migration, and invasion were promoted by miR-942-5p overexpression and inhibited by miR-942-5p downregulation. Furthermore, the reverse effect of HCG11 siRNA on cell proliferation, migration, and invasion was also found in AGS cells with miR-942-5p inhibitor (Figures 3(d)–3(f)). Collectively, miR-942-5p promotes cell proliferation, migration, and invasion by downregulating lncRNA HCG11.

### 3.4. BRMS1 is a Direct Target of miR-942-5p

**Figure 4:**
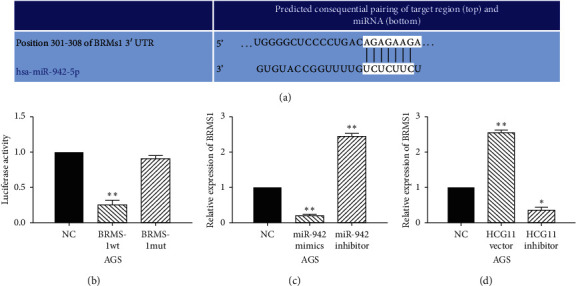
BRMS1 is a direct target of miR-942-5p. (a) The binding sites of BRMS1 and miR-942-5p. (b) Luciferase reporter assay. (c) BRMS1 expression in AGS cells with miR-942-5p mimics or inhibitor. (d) BRMS1 expression in AGS cells with HCG11 siRNA or vector.^*∗*^*P* < 0.05,  ^*∗∗*^*P* < 0.01.

Further, TargetScan (http://www.targetscan.org) predicts that miR-942-5p has a binding site on the 3′-UTR of BRMS1 (Figure 4(a)). Luciferase reporter assay indicated that miR-942-5p mimics reduced the luciferase activity of wt-BRMS1 but had no effect on mut-BRMS1 luciferase activity (Figure 4(b)). In addition, BRMS1 expression was found to be reduced by miR-942-5p mimics and promoted by miR-942-5p inhibitor in AGS cells (Figure 4(c)). On the contrary, the expression level of BRMS1 was enhanced by upregulation of HCG11 and decreased by downregulation of HCG11 in AGS cells (Figure 4(d)). These results demonstrate that BRMS1 is a direct target of miR-942-5p and can be regulated by lncRNA HCG11 in GC.

### 3.5. BRMS1 Is Involved in GC Progression by Affecting lncRNA HCG11/miR-942-5p Axis

**Figure 5:**
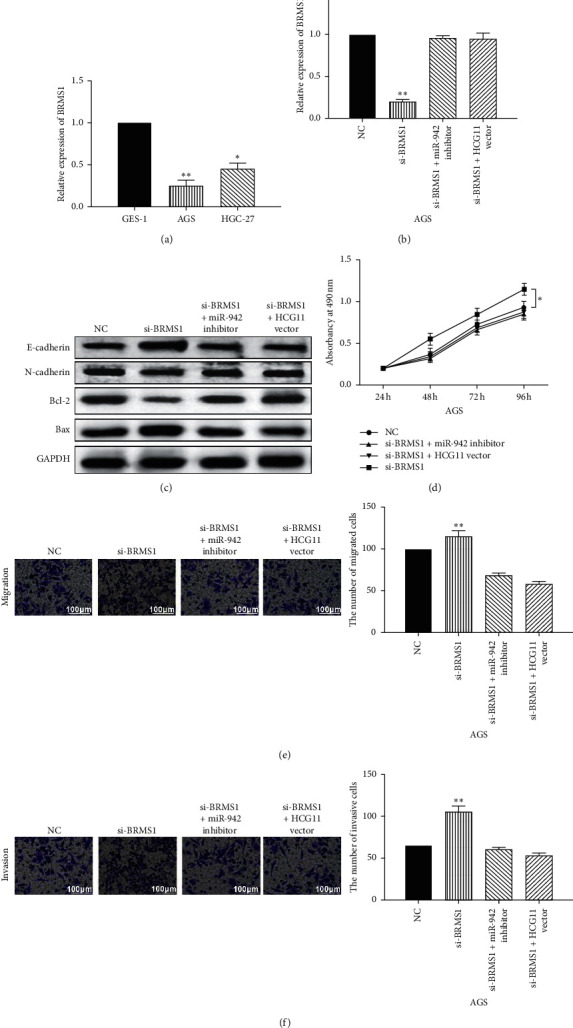
BRMS1 is involved in GC progression by affecting lncRNA HCG11/miR-942-5p axis. (a) The expression of BRMS1 was detected in GES-1, AGS, and HGC-27 cells. (b) BRMS1 expression in AGS cells with BRMS1 siRNA, BRMS1 siRNA + miR-942-5p inhibitor, or BRMS1 siRNA + HCG11 vector. (c) The protein expressions of E-cadherin, N-cadherin, Bax, and Bcl-2 in AGS cells with BRMS1 siRNA, BRMS1 siRNA + miR-942-5p inhibitor, or BRMS1 siRNA + HCG11 vector. (d, e, f) Cell proliferation, migration, and invasion in AGS cells with BRMS1 siRNA, BRMS1 siRNA + miR-942-5p inhibitor, or BRMS1 siRNA + HCG11 vector. ^*∗*^*P* < 0.05,  ^*∗∗*^*P* < 0.01.

To further explore the interaction between lncRNA HCG11/miR-942-5p axis and BRMS1, HCG11 vector or miR-942-5p inhibitor was transfected into AGS cells with BRMS1 siRNA. First, we found that BRMS1 expression was lower in AGS and HGC-27 GC cells than GES-1 cells (Figure 5(a)). After transfection of BRMS1 siRNA, BRMS1 expression was decreased in AGS cells. However, HCG11 vector or miR-942-5p inhibitor recovered the decreased expression of BRMS1 (Figure 5(b)). Moreover, BRMS1 downregulation promoted N-cadherin and Bcl-2 expressions and restrained E-cadherin and Bax expression in AGS cells. HCG11 vector or miR-942-5p inhibitor reversely regulated the effect of BRMS1 siRNA on these genes (Figure 5(c)). Besides, knockdown of BRMS1 was found to promote cell proliferation, migration, and invasion in AGS cells. Similarly, HCG11 vector or miR-942-5p inhibitor abolished the promoting effect of BRMS1 siRNA on AGS cell proliferation, migration, and invasion (Figures 5(d)–5(f)). Taken together, BRMS1 is involved in GC progression by affecting lncRNA HCG11/miR-942-5p axis.

## 4. Discussion

Recently, various lncRNAs have been demonstrated to regulate tumorigenesis of GC. For example, lncRNA LUCAT1 was upregulated in GC and promoted GC cell proliferation and invasion [19]. In addition, lncRNA MEG-3 was downregulated in GC. Overexpression of MEG-3 suppressed GC cell growth, invasion, and migration [20]. These findings suggest that lncRNAs are important regulators in GC progression. This study aimed to investigate the regulatory mechanism of lncRNA HCG11 in GC. We found that the downregulation of HCG11 expression was decreased in GC cells. Furthermore, HCG11 overexpression inhibited cell proliferation, migration, and invasion in GC. Consistent with our results, downregulation of HCG11 has been identified in prostate cancer and predicted a poor prognosis [21]. Additionally, HCG11 has been proposed to suppress the growth of glioma through cooperating with miR-4425/MTA3 axis [22]. Here, HCG11 was found to inhibit GC progression by interacting with miR-942-5p/BRMS1 axis.

It is widely recognized that lncRNAs can interact with miRNAs, thereby regulating the downstream genes. In the present study, we found that lncRNA HCG11 directly targets miR-942-5p and acts as a molecular sponge of miR-942-5p. At the same time, the role of miR-942-5p was also explored in GC. In contrast to HCG11, miR-942-5p was upregulated in GC cells. Upregulation of miR-942-5p promoted cell proliferation, migration, and invasion in GC. Similarly, Ge et al. found that miR-942-5p expression was also increased in esophageal squamous cell carcinoma and promoted cancer stem cell-like traits [23]. These findings demonstrate that miR-942-5p acts as a tumor promoter in GC. Meanwhile, we found that lncRNA HCG11 could interact with miR-942-5p in GC cells. Furthermore, HCG11 can exert a reverse effect on cell proliferation, migration, and invasion regulated by miR-942-5p in GC cells, which has not been reported in previous studies. Further, miR-942-5p was confirmed to target BRMS1 directly. Furthermore, lncRNA HCG11 can positively regulate BRMS1 expression in GC cells.

In this study, we found that BRMS1 expression was increased in GC cells. Knockdown of BRMS1 promoted cell proliferation, migration, and invasion in GC cells. In addition, BRMS1 has been found to upregulate miR-146 and suppress breast cancer metastasis [24]. Based on these results, we consider that BRMS1 plays an inhibitory effect on GC progression. Besides that, miR-346 has been demonstrated to promote migration and invasion of nasopharyngeal carcinoma cells via targeting BRMS1 [25]. Here, miR-942-5p was also found to promote GC progression by targeting BRMS1. At the same time, we also found that BRMS1 competed with HCG11 to bind with miR-942-5p, which is firstly proposed. Rescue assays showed that HCG11 inhibited GC progression by upregulating BRMS1. Collectively, lncRNA HCG11 acts as a ceRNA to regulate GC progression by mediating the miR-942-5p/BRMS1 axis. This study firstly reveals the potential involvement of HCG11 in the pathogenesis of GC and investigates its correlation with miR-942-5p/BRMS1 hitherto unreported in GC.

## 5. Conclusion

To conclude, lncRNA HCG11 inhibited cell proliferation, migration, and invasion in GC by downregulating miR-942-5p and upregulating BRMS1. Our study reveals that lncRNA HCG11 may be a potential target for GC treatments. Although the regulatory mechanism of lncRNA HCG11 has been initially elucidated in this study, the further functional mechanism of HCG11 still needs to be investigated in GC by independent cohorts and prospective trials, such as EMT and the *in vivo* study.

## Data Availability

The data used to support the findings of this study are available from the corresponding author upon request.
